# Supplementary material for: Effect of Climatic Condition, Type of Trough and Water Cleanliness on Drinking Behavior in Dairy Cows
Source: Animals (Basel). 2024 Jan 13;14(2):257. doi: 10.3390/ani14020257 (PMC10812700; doi:10.3390/ani14020257)
Supplement: Supplementary file 1 [file animals-14-00257-s001.zip › animals-2705896-supplementary.pdf]

Effect of climatic condition, type of trough and water cleanliness on drinking behavior in dairy cows: part two

Franziska Katharina Burkhardt, Jason Jeremia Hayer, Céline Heinemann, and Julia Steinhoff-Wagner

Supplementary material

Supplementary Table S1

Characteristics of the TMR presented daily at 9h in the morning and resupplied hourly by an automatic feed delivery robot and concentrate feed, fed in the milking robots over the study periods.

	TMR	Concentrate
Mean DM content (%)	87.58	33.28
Mean CP (g/kg DM)	15.26	20.9
Mean NDF (g/kg DM)	36.84	32.46
Mean ADF (g/kg DM)	19.68	11.24
Mean ADL (g/kg DM)	5.80	7.34
Mean Ash (g/kg DM)	9.29	6.41
Mean Energy (MJ	17.75	18.67

DM = dry matter; CD = crude protein; NDF = neutral detergent fiber; ADF = acid detergent fibre; ADL = acid detergent lignin GE = gross energy

Supplementary Table S2

Mean (± standard deviation) of biological and physico-chemical livestock drinking water quality variables of offered water in cold and warm ambient temperatures that were either daily cleaned or not over a period of 15 d and well water from the farm well. Samples were taken on the first and last days of the experimental periods.

Variable	Cleaned		Uncleaned		Well water	Reference value
	Cold ambient temperatures	Warm ambient temperatures	Cold ambient temperatures	Warm ambient temperatures		
Biological variables						
E. coli[2] [log_{10}(cfu/mL)]	0.1 ± 0.1	0.0 ± 0.0	0.1 ± 0.1	0.0 ± 0.0	0.0	0/100 mL[a]
CC[2] [log_{10}(cfu/mL)]	0.8 ± 0.3	1.3 ± 0.4*	0.8 ± 0.4	1.2 ± 0.3*	0.0	< 1.0[a]
TVC 20°C[3] [log_{10}(cfu/mL)]	3.8 ± 0.4	4.04 ± 0.2*	3.5 ± 0.6	4.1 ± 0.2*	1.5	< 4.0[a]
TVC 36°C[3] [log_{10}(cfu/mL)]	3.6 ± 0.3*	3.7 ± 0.3*	3.6 ± 0.2*	3.6 ± 0.3*	0.0	< 3.0[a]
Physico-chemical variables						
pH value	7.0 ± 0.0	6.8 ± 0.1	7.0 ± 0.0	6.9 ± 0.0	6.7	5–9[a]
Electrical conductivity (µS/cm)	275.3 ± 8.0	277.2 ± 1.4	268.2 ± 3.1	278.7 ± 2.0	268.0	< 3000[a]
Salinity (mg/L)	150.3 ± 4.6		143.3 ± 1.8		142.0	< 25,000[a]

Ammonium (mg/L)	0.3 ± 0.2	0.1 ± 0.1	0.09 ± 0.1	0.1 ± 0.1	0.05	< 3[a]
Chloride (mg/L)	18.3 ± 2.0	14.6 ± 0.2	18.0 ± 0.7	15.0 ± 0.3	19.0	< 500[a]
Nitrate (mg/L)	9.0 ± 1.2	0.7 ± 0.1	7.7 ± 0.4	0.6 ± 0.1	6.3	< 300[a]
Nitrite (mg/L)	0.07 ± 0.02	0.05 ± 0.0	0.05 ± 0.0	0.05 ± 0.0	0.05	< 500[a]
Sulfate (mg/L)	18.7 ± 0.5	14.0 ± 0.0	18.3 ± 0.2	13.8 ± 0.2	19.0	< 500[a]
Phosphate (mg/L)	1.1 ± 0.2		0.9 ± 0.0		0.9	-
Phosphorus (mg/L)	0.36 ± 0.1		0.3 ± 0.0		0.3	< 1000[b]
Iron (mg/L)	0.02 ± 0.0	0.02 ± 0.0	0.05 ± 0.0	0.02 ± 0.0	0.02	< 3[a]

[*] Means that exceed reference values of livestock drinking water quality.

[1] Samples were collected from cleaned and uncleaned troughs in warm and cold ambient temperatures on the first and last day of the 15-d study period.

[2] *Escherichia coli* and total coliform count (CC) measured according to DIN 38411-6 (K 6) 1991-06.

[3] Total viable count (TVC) measured according to DIN EN ISO 622 (K5) 1999-07.

[a] Based on Kamphues et al. (2007) [19].

[b] Based on Olkowski (2009) [37]

Supplementary Table S3

Description of dairy cows' drinking behavior, recorded on a commercial farm on open and valve troughs, adapted by Burkhardt et al. [10].

Variable	Unit	Description

Drinking[2] (total duration, s)	Time elapsed from the moment a cow crosses the edge of the trough until the cow leaves the trough area (ca. 1 m radius around the trough), including the beginning of the tasting period.	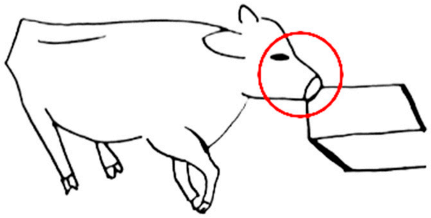 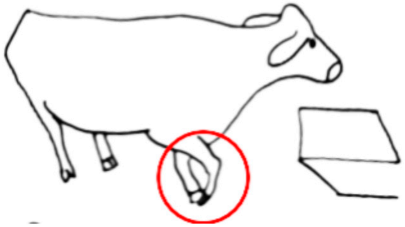

Tasting period[2] (duration, s)	s	Timespan, beginning with the cow crossing the edge of the trough and ending with water intake, including short periods of water intake < 5 sips with <3 s between sips and drinking breaks, or the cow leaving the trough.
Only tasting[1]	%	Begins with a cow crossing the edge of the trough and ending with the cows stepping away from the trough without water intake or including short periods of water intake < 5 sips excluding water intake periods >5 sips with <3 s between sips. Cows leave the trough after the tasting period.
Tasting combined with other motions[1, b]	%	**Smelling:** *Planum nasolabiale* remaining slightly above the surface of the water trough or the surface of the water without direct contact with the water. 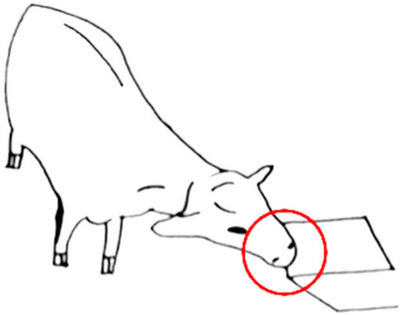 **Tasting using the tongue**: Tongue is visible outside the *planum nasolabiale. Planum nasolabiale* is above the trough area or in contact with water, but no water consumption is observed. 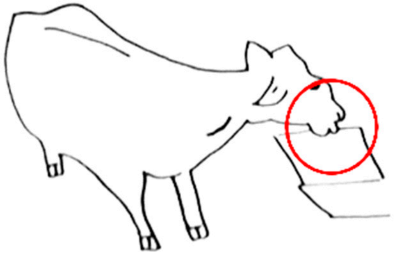

Water intake periods[2] (number)	s	"Total number of "water intake" periods during "total drinking episode", including both, water intake periods of >5 sips or during water contact with drinking breaks lasting 3 s..”	
Water intake[2, a] (duration,s)	s	Total time the *planum nasolabiale* is under the water surface or in contact with the water surface during a total drinking event, including both a water intake of >5 sips or during a water contact with drinking breaks lasting <3 s and water intake periods of <5 sips or during a water contact with drinking breaks lasting >3 s.	
Sips per drinking episode[2,a] (number)	count	Number of sips measured as counts per “drinking episode.” Total number of sips while tasting and sips per period of water intake. Sips are “a movement of the animal's throat swallowing water, while its mouth is submerged” (Filho et al., 2004 [14]) and visible by contraction of the cheek muscle and/or marked contraction of the throat and/or clear water movement while *planum nasolabiale* is in contact with the water surface.	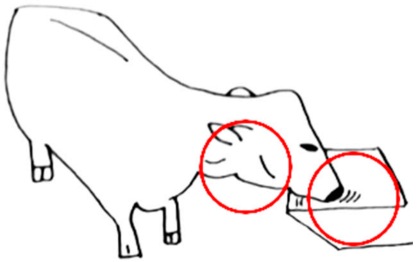

Drinking breaks[2] (number)	s	Total number of “drinking breaks” during a “the total duration of drinking.”	
Drinking breaks[2] (duration, s)	s	Total time during a drinking event the *planum nasolabiale* is above the water surface, i.e. not in contact with the water, including the time before, between and after water intake periods.	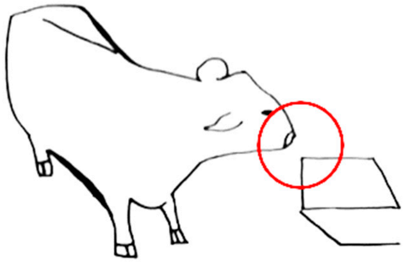
Swallowing difficulties[1]	%	Coughing with throat extended, usually combined with a visible tongue.	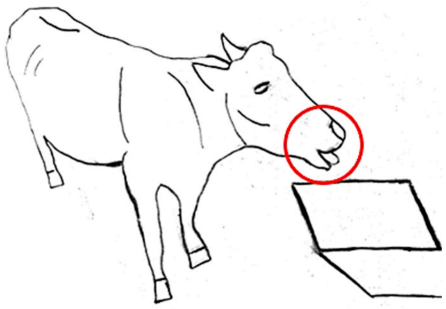
Agonistic behaviors[1]	%	Disturbance of the drinking animal by other animals or the corresponding behavior against other animals by the	

		drinking animal. Including displacement, head bump, pushing with core body.	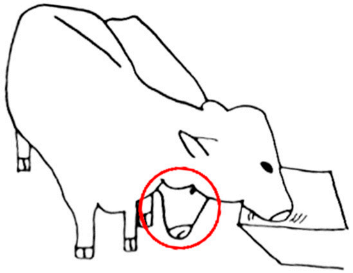
Interruption of the drinking episode due to agonistic behavior[1]	%	Interruption or termination of a drinking episode resulting from agonistic behavior.	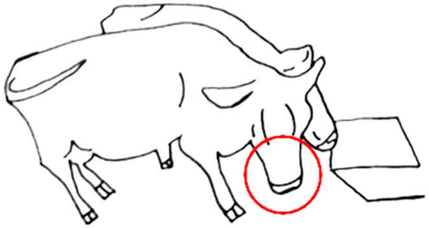

[1] continuous unit

[2] dichtomized unit

[a] Based on Kamphues et al. (2007) [19].

[b] "Smelling," "tasting using the tongue," and "looking around" after the tasting phase were not included in the evaluation.

Supplementary Table S4: Assessment of the trough cleanliness using scores (not soiled, moderate soiled, heavy soiled).

Soiling score	Not soiled (1)	Moderate soiled (2)	Heavy soiled (3)
Open troughs 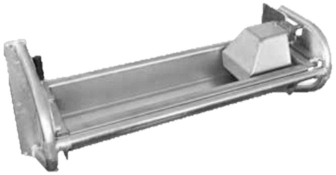	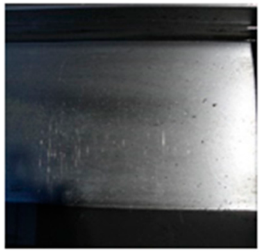	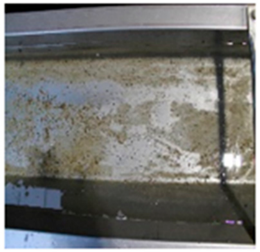	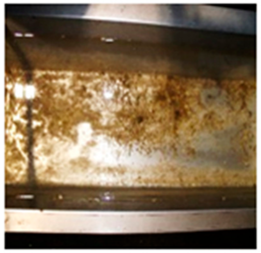
Valve troughs 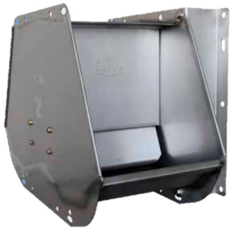	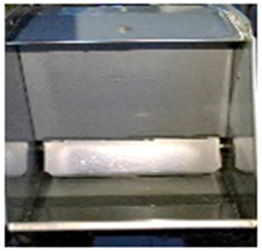	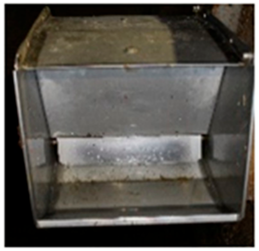	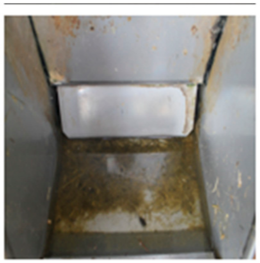

Supplementary Table S5

Dairy cow's drinking behavior in warm ambient temperatures (n = 135 cows) and in cold ambient temperatures (n = 144 cows) over a period of 15 d at two trough types (open and valve troughs) that were either daily cleaned or not. Drinking behavior variables are shown as means with standard error (SE).

Climatic condition	Trough type	Cleaning status	Total duration of drinking (s)	Duration of tasting period (s)	Drinking breaks	Duration of drinking breaks	Duration of water intake	Number of water intake periods	Number of sips per drinking episode
Warm ambient temperatures	Open trough	Daily cleaned	117.4 ± 2.5	31.9 ± 1.2	2.3 ± 0.1	47.3 ± 1.7	59.0 ± 1.2	2.4 ± 0.1	12.5 ± 0.2
		Uncleaned	106.7 ± 2.2	30.6 ± 1.1	2.1 ± 0.0	42.4 ± 1.5	55.4 ± 1.4	2.3 ± 0.1	11.7 ± 0.3
	Valve trough	Daily cleaned	140.5 ± 5.1	37.4 ± 3.1	2.4 ± 0.1	56.8 ± 3.2	72.9± 3.0	2.4 ± 0.1	12.6 ± 0.4
		Uncleaned	125.7 ± 4.3	33.8 ± 2.4	2.2 ± 0.1	49.4 ± 2.9	70.3 ± 2.7	2.3 ± 0.1	12.0 ± 0.4
Cold ambient temperatures	Open trough	Daily cleaned	110.7 ± 2.3	30.8 ± 1.1	2.6 ± 0.4	13.2 ± 0.4	27.9 ± 0.8	3.0 ± 0.1	19.7 ± 0.4
		Uncleaned	118.2 ± 2.3	32.6 ± 1.2	2.9 ± 0.1	13.9 ± 0.5	25.1 ± 0.6	3.1 ± 0.1	20.6 ± 0.5
	Valve trough	Daily cleaned	132.4 ± 3.5	32.0± 1.4	3.1 ± 0.6	14.5 ± 0.6	26.0 ± 1.1	3.1 ± 0.1	16.8 ± 0.6
		Uncleaned	136.5 ± 3.4	35.2 ± 1.5	3.1 ± 0.1	15.5 ± 0.8	28.2 ± 0.9	3.2 ± 0.1	22.3 ± 0.6
Level of significance:									
Climatic condition<br>Cleaning status			*0.1*	*0.1*	*n.a*	*<.0001*	*<.0001*	*n.a*	*<.001*

Trough Design	*0.2*	*0.9*	*n.a*	*0.07*	*0.2*	*n.a*	*0.1*
Climatic condition x Cleaning status	*<.001*	*0.3*	*n.a*	*<.001*	*0.02*	*n.a*	*<.01*
Climatic condition x Design	*0.0001*	*0.2*	*n.a*	*0.0001*	*0.02*	*n.a*	*<.001*
	0.6	*0.7*	*n.a*	*0.01*	*0.0001*	*n.a*	*0.03*
Cleaning status x Design	*0.9*	*0.4*	*n.a*	*0.8*	*0.001*	*n.a*	*<.0001*
Climatic condition x Cleaning status x Design	0.8	0.4	n.a	0.3	0.3	n.a	0.01

MSE: Standard error of mean. NS: No significant difference. SE: Standard error. N.a: Not analyzable due to infinite likelihood
